# Interpretable Predictive Modelling of Basalt Fiber Reinforced Concrete Splitting Tensile Strength Using Ensemble Machine Learning Methods and SHAP Approach

**DOI:** 10.3390/ma16134578

**Published:** 2023-06-25

**Authors:** Celal Cakiroglu, Yaren Aydın, Gebrail Bekdaş, Zong Woo Geem

**Affiliations:** 1 Department of Civil Engineering, Turkish-German University, 34820 Istanbul, Turkey; cakiroglu@tau.edu.tr; 2 Department of Civil Engineering, Istanbul University-Cerrahpasa, 34320 Istanbul, Turkey; yaren.aydin1@ogr.iuc.edu.tr; 3 College of IT Convergence, Gachon University, Seongnam 13120, Republic of Korea

**Keywords:** FRP, concrete, splitting tensile strength, machine learning, XGBoost, SHAP

## Abstract

Basalt fibers are a type of reinforcing fiber that can be added to concrete to improve its strength, durability, resistance to cracking, and overall performance. The addition of basalt fibers with high tensile strength has a particularly favorable impact on the splitting tensile strength of concrete. The current study presents a data set of experimental results of splitting tests curated from the literature. Some of the best-performing ensemble learning techniques such as Extreme Gradient Boosting (XGBoost), Light Gradient Boosting Machine (LightGBM), Random Forest, and Categorical Boosting (CatBoost) have been applied to the prediction of the splitting tensile strength of concrete reinforced with basalt fibers. State-of-the-art performance metrics such as the root mean squared error, mean absolute error and the coefficient of determination have been used for measuring the accuracy of the prediction. The impact of each input feature on the model prediction has been visualized using the Shapley Additive Explanations (SHAP) algorithm and individual conditional expectation (ICE) plots. A coefficient of determination greater than 0.9 could be achieved by the XGBoost algorithm in the prediction of the splitting tensile strength.

## 1. Introduction

Concrete is a commonly utilized construction material globally, produced by blending cement with sand, gravel, and water. Additional chemical and mineral components can be incorporated into concrete as needed [1,2]. Furthermore, the addition of fibers can enhance the mechanical properties of concrete such as the tensile strength, fatigue strength, flexural strength, impact strength, ductility, and toughness [3,4,5,6]. Since the late 1960s, fibrous concretes have been produced by the addition of discontinuous, randomly distributed fibers of different types to the concrete mix and this technology has been developing to this date [7]. Among the fiber materials used in concrete mixes, steel, glass, carbon, aramid, and various natural fiber types such as jute, sisal and bamboo can be counted [8,9,10,11,12,13,14,15].

One of the new developments in the field of fiber-reinforced concrete is the usage of basalt fiber (BF), which gives excellent results in improving the compressive and flexural strength of concrete composites. Basalt fiber has high tensile strength and ductility, and its resistance to corrosion under alkali, acidic, and salt effects is better than fiber types such as steel, carbon, and aramid, making it a suitable fiber for bridge, shoreline, and road structures [16,17]. Table 1 lists some commonly used fiber types and their corresponding properties in comparison to basalt fibers.

Previous studies have demonstrated the effectiveness of basalt fiber reinforcement in a structural engineering context. Kabay [21] studied the effect of the weight percentage and length of basalt fiber on the abrasion resistance and fracture energy. It was found that the use of basalt fiber resulted in an increase in the abrasion resistance and fracture energy even at low contents. In the study by Jin et al. [22], it was observed that the performance of basalt fiber-reinforced concrete exposed to freeze–thaw cycles was better in terms of dynamic modulus of elasticity and quality loss compared to plain concrete. Tumadhir [23] investigated the thermal and mechanical properties of basalt fiber-reinforced concrete with the different volume fractions (0.1–0.5%) of basalt fiber. The results showed that the volume fraction of basalt fibers in the concrete mixture increased the splitting tensile strength and heat resistance of the concrete. Dong et al. [24] investigated the mechanical properties and microstructures of basalt fiber with recycled aggregate concrete. Results showed that the addition of basalt fiber increased the flexural and splitting tensile strength of recycled aggregate concrete. Zhou et al. [25] conducted an experimental study on the mechanical properties of basalt fiber-reinforced concrete (0–0.6% basalt fiber by volume), and the results showed that compared to plain concrete, basalt fiber improved the performance of concrete, including toughness, crack resistance, tensile strength, and flexural strength. Jiang et al. [26] used basalt fibers with lengths of 12 and 22 mm and different volume fractions in their study. They stated that basalt fiber-reinforced concrete showed high flexural and tensile strength compared to plain specimens; and when both the volume fraction and length of the fibers increase, the engineering properties improved. Ayub et al. [27] examined the effect of basalt fibers on the mechanical properties of high-performance reinforced concrete and found that the splitting tensile strength increased by 2.7%, 5%, and 14.1% by applying 1%, 2%, and 3% bulk basalt fibers. Shen et al. [28] tested six reinforced concrete interior beam–column joints with basalt fiber-reinforced polymer sheets in different methods under the cyclic load. Results showed that strengthening with BFRP increases load-bearing capacity and ductility, and the stiffness of joints increased by sheets and improves joint energy-dissipation capability. Additionally, Bajaj [29] reported that the crack width generated in polypropylene FRC is larger than the crack width in basalt fiber-reinforced concrete. 

With the evolution of artificial intelligence (AI), machine learning (ML) algorithms are commonly used because ML can provide fast and economical solutions for civil engineering problems [30,31,32,33,34,35,36,37,38,39,40,41,42]. Moreover, in the field of fiber-reinforced composites, ML algorithms have been increasingly applied [9,43,44,45,46,47,48,49]. In what follows, some of the research carried out with basalt fibers has been summarized.

Hasanzadeh et al. [50] presented an application of ML models (Linear Regression (LR), Support Vector Regression (SVR), and Polynomial Regression (PR)) for predicting the compressive, flexural, and tensile strengths of basalt fiber-reinforced high-performance concrete (BFHPC). According to the performance metrics obtained, the PR technique gave a higher accuracy (R^2^ = 0.99) and a more reliable result than the LR and SVR algorithms. Li et al. [51] predicted the compressive strength of BFRC based on the Random Forest (RF) algorithm and compared the results with neural network and SVR methods. When the performance metrics are analyzed, it is seen that RF predicts the compressive strength of BFRC more accurately than the other models. Najm et al. [52] used statistical and ANN models to analyze and predict the effect of basalt fibers on the mechanical behavior of high-strength sustainable self-compacting concrete (SCC). The admixtures used for the production of Portland cement (i.e., ground granulated blast furnace slag (GGBS), fly ash (FA), and silica fume (SF)) were used in the design mix with different proportions of cement replacement. Basalt fibers were integrated into the concrete mixtures at 0.5%, 0.75%, and 1.00% of the total cementitious material weight. It is concluded that analysis of variance (ANOVA) and ANN techniques are efficient at analyzing, capturing and predicting the effect of different admixtures on the compressive strength of concrete, despite having uncertain behavior with experimental results. Kuang et al. [53] proposed an artificial neural network-based Back Propagation (BP) model to predict the thermal performance of a basalt fiber bundle thermal flow-reversal reactor and compared the prediction accuracy against Multiple Linear Regression (MLR) methods and Computational Fluid Dynamics (CFD) simulations. The highest success rate in prediction was observed with BP, then with MLR, and the lowest with CFD. Li et al. [54] proposed a model by combining the kernel extreme learning machine (KELM) with a genetic algorithm (GA) for predicting the compression strength of basalt fiber-reinforced concrete and compared it with four ML models (Backpropagation Neural Network (BPNN), Support Vector Regression (SVR), Gaussian Process Regression (GPR), and KELM). Their experiment confirmed that the KELM-GA model proposed in their study has better prediction performance compared to the other four machine learning algorithms. Ndepete et al. [55] investigated the effect of fiber size, fiber quantity, water content, and cell pressure on the maximum deflection stress (MDS) and failure deformation (FD) of basalt fiber (BF)-reinforced unsaturated silty soils using different intelligence techniques (artificial neural network (ANN), support vector machine (SVM) and fuzzy logic (FL)). In general, it was experimentally observed that the addition of BF increased the MDS, which generally corresponds to the shear strength of soils. The comparison of the performance metrics of the models showed that FL (R^2^ = 0.938) outperformed SVM and ANN, especially in FD prediction. Sensitivity analysis to determine the effect of inputs on MDS and FD response variables showed that fiber length and cell pressure have a significant effect on MDS predictions. Najjar et al. [56] developed an optimized artificial intelligence model consisting of Long Short Term Memory (LSTM) and Chimpanzee Optimization Algorithm (CHOA) to predict kerf quality characteristics in laser-cutting of basalt fiber-reinforced polymer composites. This model was compared with three models (stand-alone LSTM, LSTM optimized using Heap-Based Optimizer (HBO), and LSTM optimized using Manta Ray Foraging Optimization (MRFO)). According to statistical metrics, LSTM-CHOA outperformed the other three models in predicting the kerf quality characteristics of cut composites. Almohammed and Soni [57] used Random Forest (RF) and Random Tree (RT) for predicting concrete splitting tensile strength using basalt fiber-reinforced concrete. Random Forest showed the best accuracy for predicting the splitting tensile strength according to performance metrics. 

In this study, ensemble learning techniques such as Extreme Gradient Boosting (XGBoost), Light Gradient Boosting Machine (LightGBM), Random Forest, and Categorical Boosting (CatBoost) have been applied to the prediction of the splitting tensile strength of concrete reinforced with basalt fibers. A dataset of 135 splitting tensile strength experiments has been curated from the literature. Based on this dataset, the splitting tensile strength has been predicted as a function of 12 different independent input features. Furthermore, the impact of each input feature on the model predictions has been quantified using SHAP analysis and individual conditional expectation (ICE) plots. The features have been ranked according to their importance, and the most impactful features have been determined. The introduction of basalt fibers into concrete is a relatively recent development in structural engineering, and there is a lack of reliable techniques for the prediction of basalt fiber-reinforced-concrete strength. The current study aims at developing novel data-driven methodologies for the prediction of the splitting tensile strength of concrete reinforced with basalt fibers. Due to the well-known weakness of concrete under tensile forces, and the increasing application of fiber-reinforced concrete, the availability of sound methodologies for the assessment of basalt fiber-reinforced concrete tensile strength bears great significance. 

## 2. Methods

This section presents the statistical properties of the dataset used in training the machine learning models in detail. The original dataset collected from the literature [27,58,59,60,61,62,63,64,65,66,67,68,69,70,71,72,73,74,75] consisted of 205 samples. However, in order to minimize the amount of missing features the number of samples has been reduced to 135. This dataset has been split into a training set and a test set in a 70% to 30% ratio. This split ratio is adopted by a large number of ML studies and shown to be effective by the comprehensive study of Nguyen et al. [76], which compared the results obtained from nine different dataset split ratios. The data preprocessing phase also includes the scaling of the training and test sets. The MinMaxScaler function of the Scikit-learn library version 1.0.2 was used for the scaling of the dataset. The MinMaxScaler function maps every input feature into the range of [0, 1] by default such that each input feature has the maximum value of 1. The current study also utilized the k-fold cross-validation approach in training the ML models. In this approach, the training set is split into k disjoint subsets and the models are trained using k-1 of these subsets, while the k-th subset is used for testing the model performance. In this study, k is equal to 10. Furthermore, the hyperparameters of each ML model have been optimized using the GridSearch function available in the scikit-learn package available for the Python programming language. In Section 2.1, the correlation plots and ranges of the features are visualized. The equations for the prediction of the splitting tensile strength are presented in Section 2.2. A brief theoretical background for the machine learning algorithms has been provided in Section 2.3.

### 2.1. Statistical Properties of the Dataset

**Figure 1:**
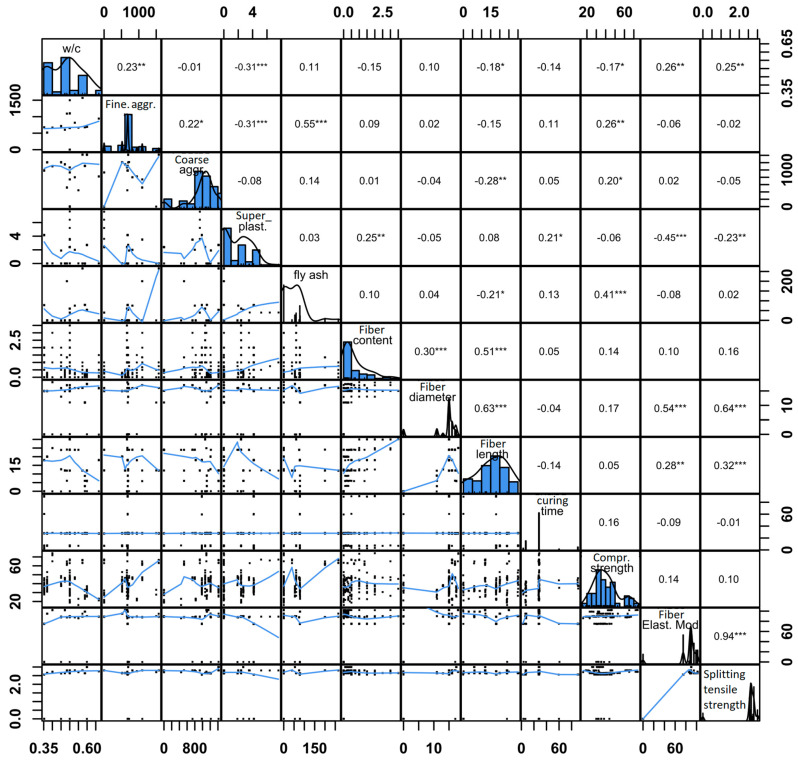
Correlation plot of the dataset.

Figure 1 shows the correlation plot of all the features included in the dataset. The diagonal of Figure 1 shows histograms and density plots of the features, whereas the upper triangular part contains the Pearson correlation coefficients between different features. The calculation of the Pearson correlation coefficient $r_{\mathrm{xy}}$ is shown in Equation (1), where x and y are two data series of equal length and n is the length of these series.

(1) $$r_{\mathrm{xy}}=\frac{n\sum_{i=1}^{n}x_{i}y_{i}-\sum_{i=1}^{n}x_{i}\sum_{i=1}^{n}y_{i}}{\sqrt{n\sum_{i=1}^{n}x_{i}^{2}-{\left(\sum_{i=1}^{n}x_{i}\right)}^{2}}\sqrt{n\sum_{i=1}^{n}y_{i}^{2}-{\left(\sum_{i=1}^{n}y_{i}\right)}^{2}}}$$

The stars in the upper right of the correlation values in Figure 1 indicate the strength of correlation between two variables. A strong correlation can be interpreted as an indicator of feature importance in the process of feature selection in the data preprocessing phase. The lower left triangular part of the correlation plot shows bivariate scatter plots of the features. The range of each feature is indicated in a horizontal and a vertical axis. According to Figure 1, the strongest linear correlation exists between the splitting tensile strength and the fiber elastic modulus with a correlation coefficient of 0.94. Moreover, a strong correlation exists between splitting tensile strength and the fiber diameter (0.64). The strongest inverse correlation indicated by a negative correlation coefficient exists between the amount of superplasticizer in the mix and the water-to-cement ratio (w/c) with $r_{\mathrm{xy}}=-0.31$. The same magnitude of inverse correlation also exists between the amounts of superplasticizer and fine aggregate.

**Figure 2:**
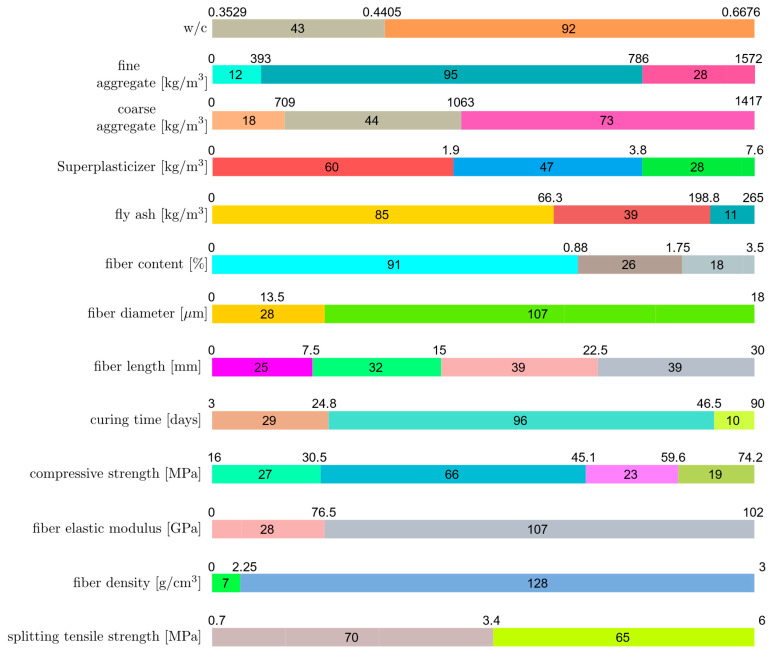
Feature ranges.

The range of each feature in the model is shown in Figure 2 with horizontal bars. The feature ranges are split into intervals of equal size, and the length of each interval in Figure 2 corresponds to the number of samples that belong to that interval. The upper and lower bounds of each interval have been written above the interval boundaries. The first horizontal bar in Figure 2 shows the distribution of the water-to-cement ratio (w/c). In this first horizontal bar, the entire range of the w/c values has been split into two equally sized intervals between 0.35 and 0.67. There are 43 samples with 0.35≤w/c≤0.44 and 92 samples with 0.44≤w/c≤0.67. The second horizontal bar in Figure 2 shows the range of the fine aggregate amount in the mix. The entire range of this input feature has been split into four intervals of equal length ($393\;\mathrm{kg}/m^{3}$). However, since the last interval contains only 5% of the samples in the entire dataset, the last two intervals have been merged. Similarly, for fiber content and curing time, the last two intervals have been merged due to the small number of samples in these intervals. It can be observed that 79% of the samples have a fine aggregate content of less than 786 $\mathrm{kg}/m^{3}$. In the case of fiber length, the range of this feature has been split into four intervals of equal size, and all of these intervals have been shown in Figure 2, since the samples are evenly distributed into these intervals and all intervals have a comparable number of samples in them. The zero lower bounds for the fiber diameter, fiber elastic modulus, and fiber length indicate the existence of test samples without fiber content.

### 2.2. Equations for the Prediction of the Splitting Tensile Strength

The relationship between the fiber content ($V_{f}$ [%]) of the fiber additive affecting the splitting tensile strength of BFRC and the tensile strength obtained from the study of Siddiqui and Sawant [77] are given in Equations (2) and (3) for 7 and 28 days, respectively. In Equations (2) and (3), $F_{t}$ represents the splitting tensile strength and $V_{f}$ represents percentage fiber content. It is understood from Equations (2) and (3) that basalt fiber additives in different ratios have a significant effect on the splitting tensile strength. At the same time, the splitting tensile strengths of BFRC for 7 days and for 28 days with the same fiber content also differ from each other. While basalt fiber concretes are evaluated, besides the ratio, the day values have an important role and should be examined in terms of these values. The splitting tensile strength of reinforced concrete increases up to a certain value of basalt fiber ratio. The fiber content giving the maximum value of splitting tensile strength (2.61 MPa and 3.68 MPa) for both equations is 1.5%. The splitting tensile strength of reinforced concrete increases up to a certain value of basalt fiber ratio. The fiber content used should also not be more than an optimum value. Above a value of 1.5%, the splitting tensile strength drastically decreases.

(2) $$F_{t}=-0.1307\times V_{f}{}^{2}+0.552\times V_{f}+1.9271$$

(3) $$F_{t}=-0.3629\times V_{f}{}^{2}+1.171\times V_{f}+2.2133$$

### 2.3. Ensemble Learning Methods

The ensemble learning approach combines the predictions of single learning models such as single decision trees to obtain strong learner models. Ensemble learning methods such as eXtreme Gradient Boosting (XGBoost), Categorical Boosting (CatBoost), Light Gradient Boosting Machine (LightGBM), and Random Forest have been successfully applied in the area of engineering systems response prediction. The output of ensemble learning algorithms can be summarized as in Equation (4) where $f_{k}\left(x\right)$ represents the output of a single decision tree for a data sample x, N is the total number of decision trees in the model, and $f\left(x\right)=\hat{y}$ is the prediction of the ensemble learning model for the input vector **x**.

(4) $$f\left(x\right)=\overset{N}{\underset{k=1}{\sum }}f_{k}\left(x\right)=\hat{y}$$

(5) $$\mathrm{Obj}=\overset{n}{\underset{i=1}{\sum }}L\left(y_{i},{\hat{y}}_{i}\right)+\overset{N}{\underset{k=1}{\sum }}\Omega \left(f_{k}\right)$$

The ensemble learning models aim to minimize the error between the predictions and the actual values which can be represented as in Equation (5), where L is the loss function, n is the total number of samples in the training set, Ω is the regularization term which prevents overfitting and enhances model performance on new datasets, and Obj denotes the objective function being minimized [78,79,80].

## 3. Results

The predictions of the equations from the literature as well as the ensemble learning techniques are presented in the following section. The predictions are compared with the experimental measurements, and the accuracies of different modeling techniques are quantified using coefficient of determination, root mean squared error and mean absolute error. The calculations of these error metrics are given in Appendix A.

### 3.1. Predictive Equations

**Figure 3:**
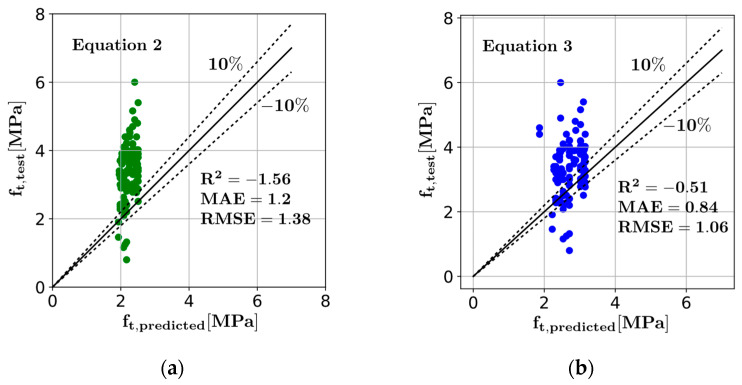
Comparison of the predictive equations (**a**) Equation (2), (**b**) Equation (3).

The predictions of Equations (2) and (3) have been presented in Figure 3, where the diagonal straight line indicates a perfect match between the predicted and actual values and the dotted lines indicate ±10% deviation from the perfect match. It can be observed that the equations significantly underpredict the measured splitting tensile strength values. Furthermore, the negative $R^{2}$ scores for both equations indicate that the equation performance is worse than using the average value as the prediction. Moreover, the mean absolute error (MAE) and root mean squared error (RMSE) values of the equations are significantly greater than the machine learning models presented in the next section.

### 3.2. Ensemble Learning Techniques

**Figure 4:**
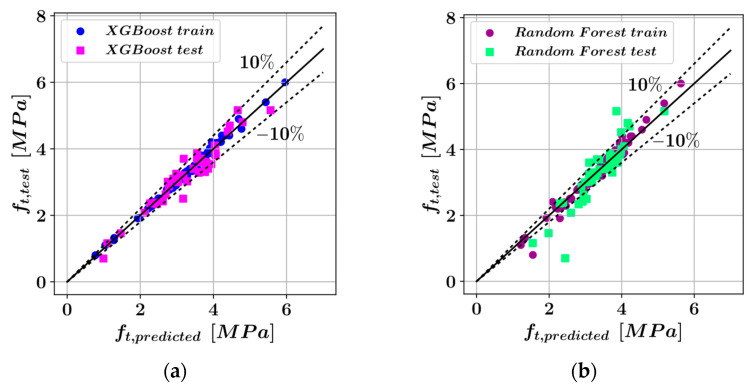

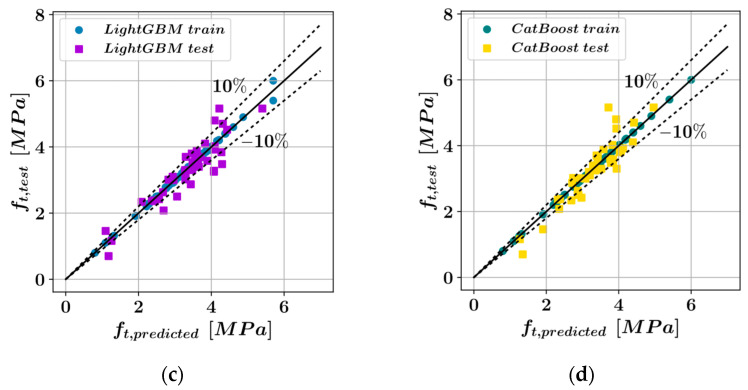
Comparison of the predicted and actual splitting tensile strength values using (**a**) XGBoost, (**b**) Random Forest, (**c**) LightGBM, and (**d**) CatBoost.

The predictions of the ensemble learning models have been plotted against the actual measurements in Figure 4. Similar to Figure 3, the perfect match between the measured and predicted values and ±10% deviations have been represented with straight and dotted lines. Each sample of the dataset has been shown with a colored dot in Figure 4. Furthermore, the training and test set members are shown with different colors. It can be observed that most data samples stayed within the ±10% deviation lines.

**Figure 5:**
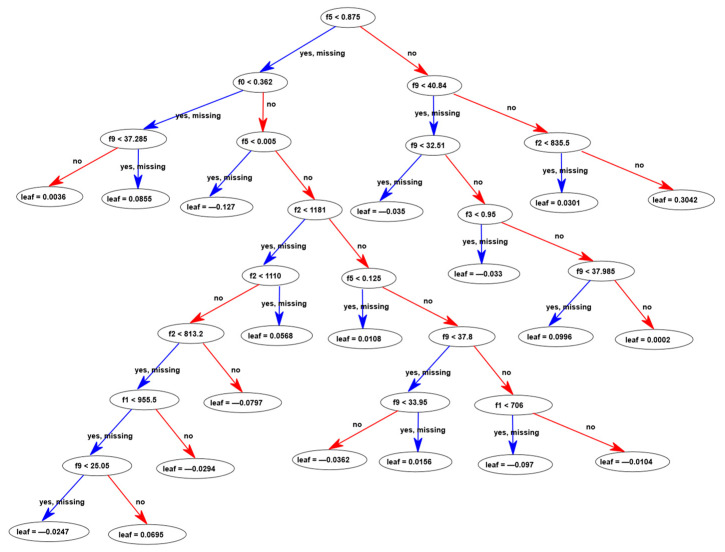
Fourth decision tree of the XGBoost model.

The ensemble models are combinations of iteratively generated decision trees. Figure 5 shows the fourth decision tree taken from the XGBoost model, which consists of a total of one hundred decision trees. In the decision tree shown in Figure 5, each node is split at a certain value of an input feature into two branches depending on the value of the input feature. Figure 5 shows that the algorithm can also handle missing values for an input feature. The input features denoted with $f_{0}$, $f_{1}$, $f_{2}$, $f_{3}$, $f_{5}$, $f_{9}$ correspond to the water-to-cement ratio, amounts of fine aggregate, coarse aggregate, superplasticizer, fiber content, and compressive strength, respectively. For any given data sample, the decision tree reaches a particular leaf node and the value in that leaf node becomes the contribution of that decision tree to the overall ensemble model prediction for the splitting tensile strength. A negative value in a leaf node indicates a decreasing corrective contribution from the decision tree, whereas a positive value indicates an increasing contribution.

**Figure 6:**
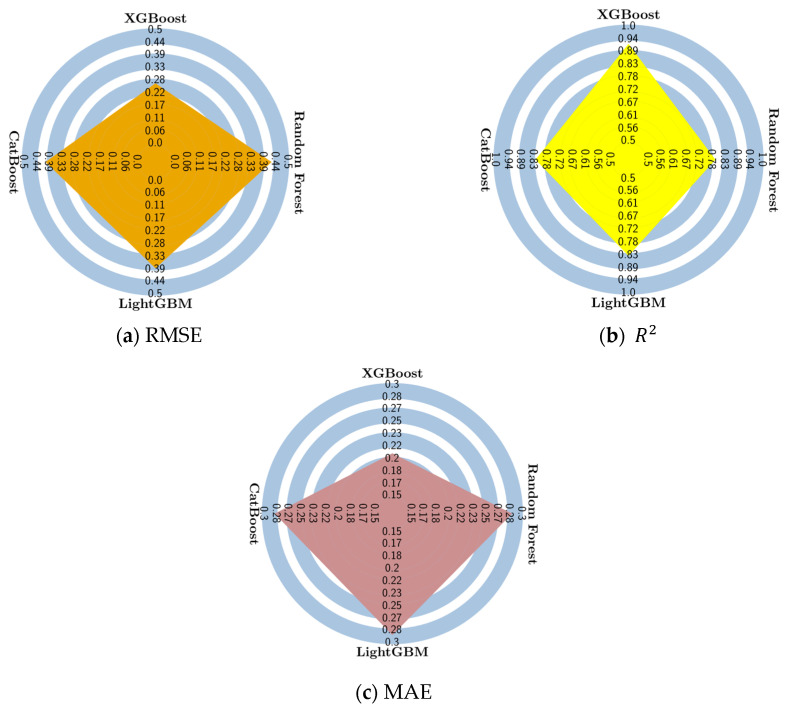
Radar plots showing the accuracy with respect to (**a**) root mean squared error (RMSE), (**b**) coefficient of determination ($R^{2}$), (**c**) mean absolute error (MAE).

**Figure 7:**
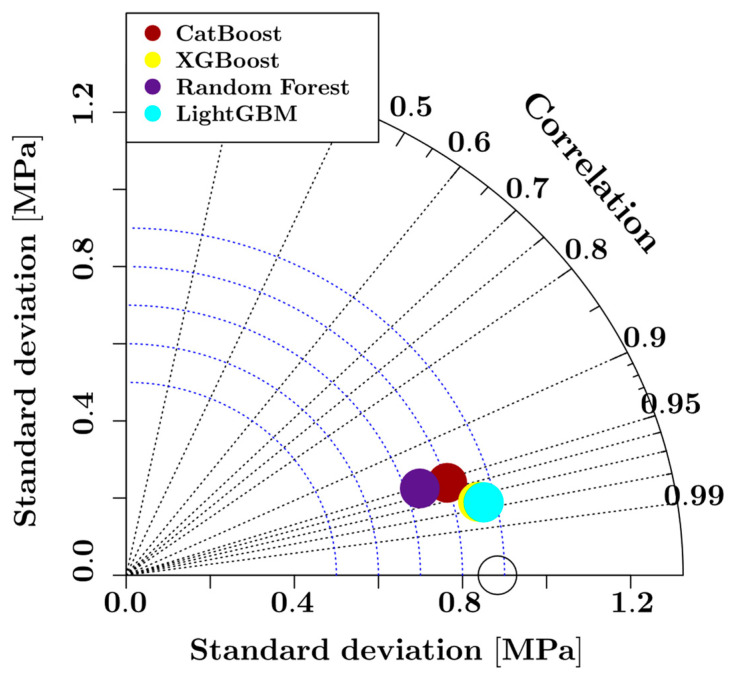
Taylor diagram of the ML models.

The accuracies of the ensemble learning models are visualized in radar chart form in Figure 6 for the test set. The numerical values for the model performance indicators are also listed in Table 2. In addition to the error metrics listed in Figure 6 and Table 2, the Pearson correlation values of each predictive model have also been visualized in a Taylor diagram in Figure 7. According to Figure 6 and Table 2, the XGBoost model performs best on the test set in terms of all three metrics. Table 2 also lists the duration of training and testing each model where the duration of the 10-fold cross-validation process is also included. According to these duration values, the Random Forest model delivered the fastest results, whereas the CatBoost model was the slowest. It should be noted that the duration of the hyperparameter tuning with grid search is not included in the duration values in Table 2. According to Figure 7, all model predictions were highly correlated with the experimental values with the Pearson correlation coefficient exceeding 0.95. The Random Forest and CatBoost models have a Pearson correlation of 0.95, whereas the XGBoost and LightGBM models have a Pearson correlation of 0.97 with respect to the entire dataset. In addition to the Pearson correlation coefficients shown on the radial axis, Figure 7 also presents the standard deviation of each model with respect to the experimentally measured splitting tensile strength values on the horizontal and vertical axes. Figure 7 shows that the Random Forest model has the least standard deviation, of around 0.72, whereas the CatBoost model has a standard deviation of 0.8. The XGBoost and LightGBM models have a standard deviation of around 0.86. The optimal hyperparameters for each ensemble model have been explored using the grid search approach which is implemented in the scikit-learn library. The optimal values and the search spaces for each hyperparameter analyzed in the grid search process are listed in Table 3. 

### 3.3. SHAP Analysis

The SHAP methodology effectively visualizes the impact of each input feature on the predictions of an ML model. This technique has its origin in cooperative game theory, where the Shapley values are used to measure the contribution of each player to a coalition. The Shapley values $\phi_{i}$, which are the basis of this methodology, can be calculated as in Equation (6), where *F* is the set of all input features and *S* is a subset of *F* which does not contain the feature with index *i*. According to Equation (6), the impact of a feature is determined by the differences in the model outputs when a feature is included and withheld from the set of input features [81,82,83].

(6) $$\phi_{i}=\underset{S\subseteq F\backslash \left\{i\right\}}{\sum }\frac{\left|S\right|!\left(\left|F\right|-\left|S\right|-1\right)!}{\left|F\right|!}\left[f_{S\cup \left\{i\right\}}\left(x_{S\cup \left\{i\right\}}\right)-f_{S}\left(x_{S}\right)\right]$$

**Figure 8:**
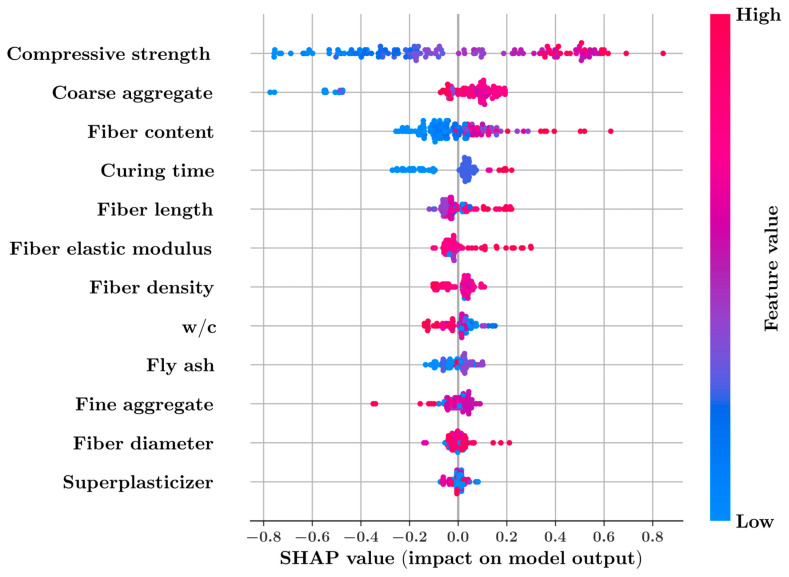
SHAP summary plot for the XGBoost model.

The SHAP summary plot in Figure 8 ranks each input feature with respect to its impact on the output of the XGBoost model. In Figure 8, every data sample is represented with a dot colored in shades of red and blue corresponding to high and low values of a feature, respectively. The horizontal position of a sample is related to the increasing or decreasing effect of a feature on the model prediction in that particular sample. In data samples located on the positive side of the SHAP values in the summary plot, the addition of the corresponding input feature to the set of all features has an increasing effect on the model prediction. 

**Figure 9:**
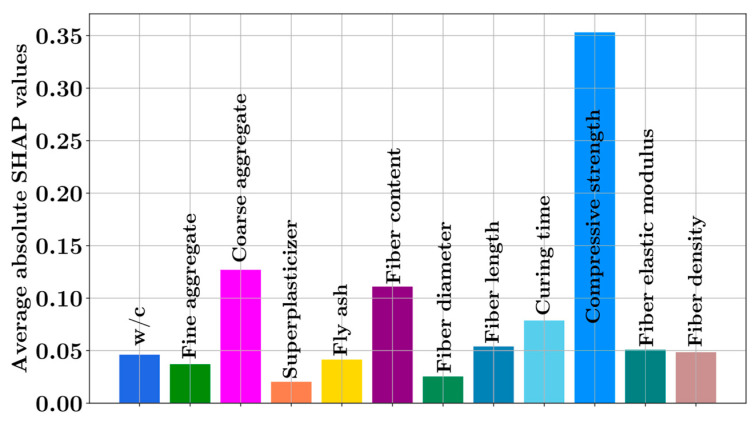
Average absolute SHAP values for the input features.

Figure 8 shows that compressive strength is the most impactful feature in predicting the splitting tensile strength, followed by the amount of coarse aggregate, fiber content, and curing time. It can be observed that increasing the compressive strength, amount of coarse aggregate, fiber content, and curing time has an increasing effect on the model predictions. On the other hand, increasing the water-to-cement ratio has a decreasing effect on the model prediction. Figure 9 ranks the input features with respect to their feature importance. The feature importance values are obtained by the average absolute SHAP values of the features. According to Figure 9, the compressive strength is the most decisive parameter, followed by the amount of coarse aggregate and the fiber content.

**Figure 10:**
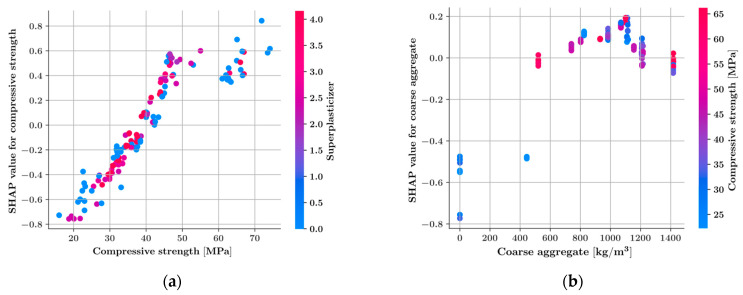

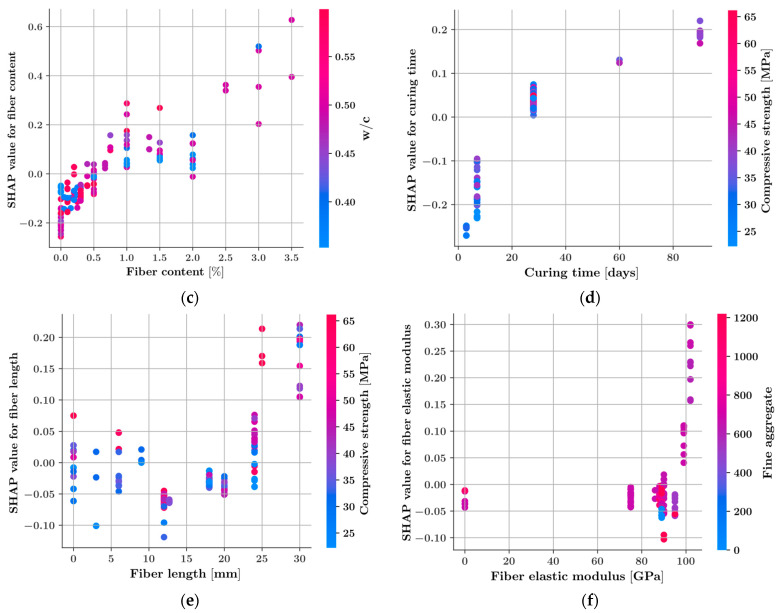
Feature dependence plots (XGBoost) for (**a**) compressive strength, (**b**) the amount of coarse aggregate, (**c**) fiber content, (**d**) curing time, (**e**) fiber length, (**f**) fiber elastic modulus.

Figure 10 shows the feature dependence plots for the six most impactful input features. Each feature dependence plot in Figure 10 shows the variation of the SHAP values of an input feature, as this input feature takes a range of values. The colors of the dots in the feature dependence plots represent the values of another most dependent input feature. Figure 10a shows that as the compressive strength increases, the SHAP value increases as well, which indicates the increasing effect of the compressive strength on the model output. Moreover, it should be noted that as the compressive strength values exceed 50 MPa, the variation of the SHAP values becomes irregular, and no clear increase can be observed. Figure 10b shows that increasing the amount of coarse aggregate in the mixture up to 1100 kg/$m^{3}$ has an increasing effect on the tensile strength. However, exceeding this amount adversely effects the tensile strength. Figure 10c,d show that the fiber content and curing time have an increasing effect on the tensile strength.

### 3.4. Individual Conditional Expectation (ICE) Plots

**Figure 11:**
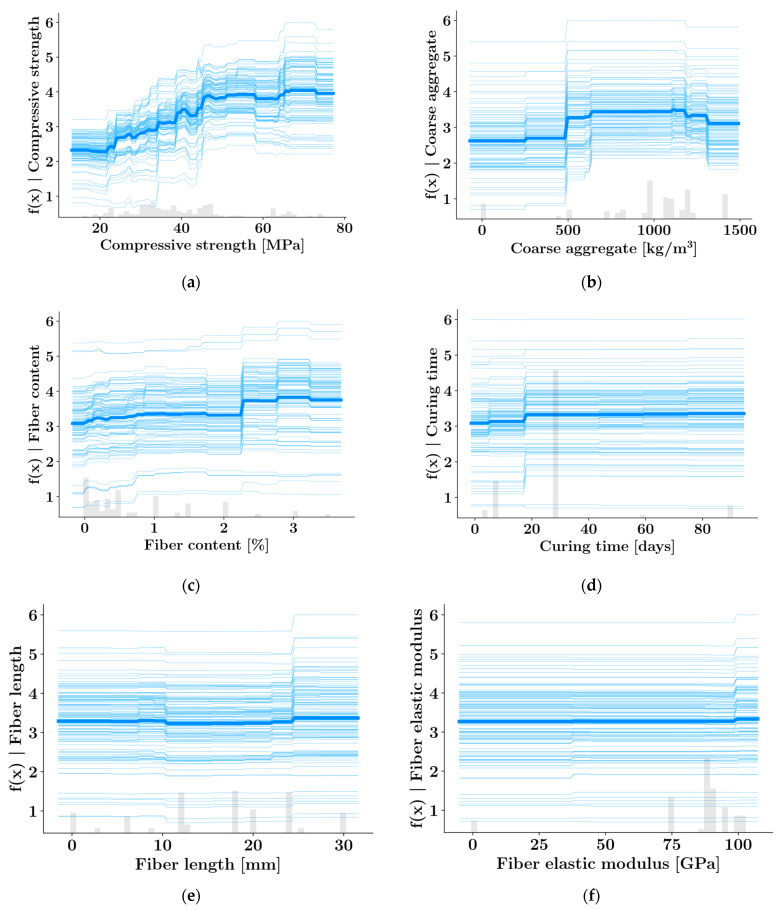
Individual conditional expectation (ICE) plots (XGBoost) for (**a**) compressive strength, (**b**) the amount of coarse aggregate, (**c**) fiber content, (**d**) curing time, (**e**) fiber length, (**f**) fiber elastic modulus.

The individual conditional expectation plots in Figure 11 display the variation of the XGBoost model output with respect to the six most impactful features. Each individual curve in Figure 11 corresponds to one of the data samples. The values of every other input feature are kept fixed in each of these curves while the variation with respect to one of the input features is plotted. The average value of these curves is plotted as a thick blue line in Figure 11. Figure 11a confirms that the splitting strength rises with compressive strength. It should be noted that the increasing effect of the compressive strength is more pronounced up to around 50 MPa. For compressive strength values greater than 50 MPa, the tensile strength values do not increase significantly. Figure 11b shows that the average tensile strength increases up to a certain amount of coarse aggregate. For coarse aggregate amounts greater than 1100 $\mathrm{kg}/m^{3}$, the tensile strength begins to decrease. The fiber content and curing time have a slightly increased effect on the tensile strength according to Figure 11c,d while the tensile strength stays mostly constant as the values of fiber length and fiber elastic modulus increase.

## 4. Discussion and Conclusions

Basalt fibers are finding applications in structural engineering in recent years for reinforcing concrete. The splitting tensile strength is one of the decisive parameters that define the strength of concrete. However, the currently available equations for the prediction of this quantity are not sufficiently developed to include the effects of a large number of variables. The current study presents an effective way of predicting the splitting tensile strength of basalt fiber-reinforced concrete with reasonable accuracy based on data obtained from experiments. A total of 135 experimental results were used in training and testing four different ensemble learning algorithms. Among these algorithms, XGBoost delivered the highest accuracy after hyperparameter tuning with an $R^{2}$ score of 0.92 on the test set. Overall, an average $R^{2}$ score of 0.85 could be achieved by the ML models. The performance of some of the currently available predictive equations in the literature was also investigated. The accuracy of these predictive equations was found to be significantly less than the ensemble learning algorithms. In terms of computational speed, the Random Forest algorithm delivered the fastest result, while the XGBoost and LightGBM algorithms also demonstrated similar speed. The CatBoost algorithm was significantly slower than these algorithms. In addition to model performances, the impact of each input feature on the model predictions was also investigated using the SHAP methodology. According to the SHAP analysis, the compressive strength was the most impactful variable in predictions of the splitting tensile strength. The second, third, and fourth most impactful features were found to be the amount of coarse aggregate, fiber content, and curing time. These four features were also found to have an increasing effect on the model predictions, while the water-to-cement ratio was found to have a decreasing effect on the predicted splitting tensile strength values. To have a better understanding of the effect of different features on the model output, individual conditional expectation plots were generated. These plots showed a slight decrease in the tensile strength when the amount of coarse aggregate in the mix exceeds 1100 kg/$m^{3}$. 

A limitation of the current study is the size of the dataset used in training and testing the ML models. In order to develop predictive models that could be reliably generalized, large datasets are necessary. Moreover, it should be noted that the database used in the study consists of experimental results with small-scale specimens, and size–scale effects are built into the results. Future research in this area may focus on obtaining larger data sets. These larger datasets could also be used for developing reliable closed-form predictive equations for the splitting tensile strength. Furthermore, additional mechanical properties of basalt fiber-reinforced concrete, such as flexural strength, could be investigated.

## Figures and Tables

**Table 1:** Typical properties of fibers [6,16,17,18,19,20].

Fiber Type	Relative Density (g/cm^3^)	Diameter (µm)	Tensile Strength (MPa)	Modulus of Elasticity (MPa)	Strain at Failure (%)
Basalt	2.60–2.65	6–25	2800–3000	70,000–90,000	3.1
Steel	7.80	100–1000	500–2600	210,000	0.5–3.5
Glass					
E*	2.54	8–15	2000–4000	72,000	3.0–4.8
AR*	2.70	12–20	1500–3700	80,000	2.5–3.6
Synthetic					
Acrylic	1.18	5–17	200–1000	17,000–19,000	28–50
Aramid	1.44	10–12	2000–3100	62,000–120,000	2–3.5
Carbon	1.90	8–0	1800–2600	230,000–380,000	0.5–1.5
Nylon	1.14	23	1000	5200	20
Polyester	1.38	10–80	280–1200	10,000–18,000	10–50
Polyethylene	0.96	25–1000	80–600	5000	12–100
Polypropylene	0.90	20–200	450–700	3500–5200	6–15
Natural					
Wood cellulose	1.500	25–125	350–2000	10,000–40,000	
Sisal			280–600	13,000–25,000	3.5
Coconut	1.12–1.15	100–400	120–200	19,000–25,000	10–25
Bamboo	1.50	50–400	350–500	33,000–40,000	
Jute	1.02–1.04	100–200	250–350	25,000–32,000	1.5–1.9
Elephant grass		425	180	4900	3.6

AR*: Alkali-resistant; E*: Electrical glass.

**Table 2:** Model accuracies in predicting the splitting tensile strength.

Algorithm	R^2^		MAE		RMSE		Duration [s]
	Train	Test	Train	Test	Train	Test	
XGBoost	0.9940	0.9228	0.0459	0.2072	0.0659	0.2643	5.94
Random Forest	0.9670	0.7960	0.1069	0.2877	0.1546	0.4296	4.07
LightGBM	0.9968	0.8361	0.0211	0.2900	0.0479	0.3850	5.19
CatBoost	0.9981	0.8222	0.0281	0.2850	0.0364	0.4010	28.07

**Table 3:** Hyperparameters for the ensemble learning models.

Model	Parameter	Grid Search Range	Value
Random Forest	n_estimators	[100, 300, 1000]	1000
-	bootstrap	[True, False]	True
-	min_samples_split	[1, 5, 10]	5
-	min_samples_leaf	[1, 5, 10]	1
-	max_features	[auto, sqrt, log2]	auto
XGBoost	colsample_bytree	[0.1, 0.3, 0.5, 1.0]	0.5
-	gamma	[0, 10, 20]	0
-	learning_rate	[0.03, 0.3, 0.5, 0.9]	0.9
-	max_depth	[2, 4, 6, 8,12]	8
-	min_child_weight	[3, 10, 20, 40, 80, 400]	3
-	reg_alpha	[0, 10, 20]	0
-	reg_lambda	[0, 10, 20]	10
LightGBM	n_estimators	[100, 200, 300]	300
-	colsample_bytree	[0.1, 0.3, 0.5, 1.0]	1.0
-	boosting_type	[gbdt, rf, dart]	gbdt
-	num_leaves	[5, 10, 20, 40]	10
-	max_depth	[1, 3, 5, None]	3
-	learning_rate	[0.2, 0.4, 0.6, 0.9]	0.6
CatBoost	iterations	[500, 1000, 3000]	1000
-	leaf_estimation_method	[Newton, Gradient, Exact]	Gradient
-	depth	[6, 8, 10]	8
-	max_leaves	[16, 64, 256]	256
-	learning_rate	[0.03, 0.3, 0.5, 0.9]	0.5
-	bootstrap_type	[Bayesian, Bernoulli, MVS]	MVS

## Data Availability

The dataset used in this paper is available under the GitHub link https://github.com/ccakiroglu/BFRC.

## Appendix A

Definitions of the accuracy metrics used in this paper, where $P_{i}$ and ${\hat{P}}_{i}$ stand for the actual and predicted values of a variable, respectively:

(A1) $$R^{2}=1-\frac{\sum_{i=1}^{N}{\left(P_{i}-{\hat{P}}_{i}\right)}^{2}}{\sum_{i=1}^{N}{\left(P_{i}-P_{\mathrm{mean}}\right)}^{2}}$$

(A2) $$\mathrm{RMSE}=\sqrt{\frac{\sum_{i=1}^{N}{\left(P_{i}-{\hat{P}}_{i}\right)}^{2}}{N}}$$

(A3) $$\mathrm{MAE}=\frac{\sum_{i=1}^{N}\left|P_{i}-{\hat{P}}_{i}\right|}{N}$$
